# Dexamethasone Induced Sino-Orbital Mucormycosis In a Patient Infected With COVID-19

**DOI:** 10.22088/cjim.13.0.277

**Published:** 2022

**Authors:** Masoud Mardani, Ilad Alavi Darazam, Abdolreza Babamahmoodi

**Affiliations:** 1 Infectious Diseases and Tropical Medicine Research Center, Shahid Beheshti University of Medical Sciences, Tehran, Iran

**Keywords:** Severe acute respiratory syndrome coronavirus 2, COVID-19, Tomography, Spiral computed, Pneumonia.

## Abstract

**Background:**

The most common causes of immunodeficiency are iatrogenic and the result of the widespread use of therapies which modulates the immune system, whether they are planned or haphazardly. Mucormycosis is an invasive fungal disease which is usually secondary to immunosuppression, diabetic ketoacidosis, and long-term use of antibiotics, corticosteroids, and cytotoxic drugs. There are researches which show patients with coronavirus disease 2019 (COVID-19), especially severely ill or immunocompromised, are more likely to suffer from invasive fungal infections. Patients with diabetes are at a higher risk for severe COVID-19 outcomes. However, there has been no clear evidence on the relationship between pre-diabetes state and mucormycosis as a complication of SARS-CoV-2 infection so far.

**Case Presentation:**

Here, we report a case of sino-orbital mucormycosis in a pre-diabetic 54-year-old female without any underlying diseases. The patient suffered from COVID-19 pneumonia. She received 8 mg dexamethasone for 12 days. Afterwards, she returned three days after her discharge with a complaint of pre-orbital cellulitis, unilateral facial numbness and decreased visual acuity. Therefore, after primary diagnostic imaging, she was regarded as a candidate for invasive surgical intervention and was consequently treated with a combination of liposomal amphotericin B, radical recurrent surgery and posaconazole.

**Conclusion:**

It is very important to consider patients who are in the pre-diabetic state or possibly immunocompromised before prescribing steroids. The patients should be examined for invasive fungal infections in post-discharge period.

The emergence of severe acute respiratory syndrome coronavirus 2 (SARS-CoV-2) has caused a large global outbreak which is regarded as a main global health problem at the moment (1). The disease varies from asymptomatic or mild infection to critical illness with acute respiratory distress syndrome. Opportunistic infections following respiratory viral infections have been known since the influenza outbreak in 1918 (2). Other respiratory viruses, including parainfluenza virus and respiratory syncytial virus, have similarly predisposed patients to invasive fungal diseases (3). Aspergillus and Candida are the main pathogens for fungal co-infections in patients with severe coronavirus disease 2019 (COVID-19). Other rare opportunistic pathogenic fungi that cause concomitant infections should also be considered (i.e., Mucormycosis and Cryptococcus) (4). Mucormycosis is a rare fatal invasive fungal disease that affects the paranasal sinuses, orbit, central nervous system, and other organs. 

This infection is usually secondary to immunosuppression, diabetes mellitus, particularly with ketoacidosis and long-term use of antibiotics, solid organ transplantation, as well as corticosteroids and cytotoxic drugs (5). COVID-19 patients with trauma, diabetes mellitus, hemopoietic malignancy, long-term neutropenia, allogeneic hematopoietic stem cell transplant, solid organ transplant, and those who use corticosteroids are more likely to develop mucormycosis (5). The disease management includes treatment of the underlying diseases and surgical removal in the combination of antifungal agents (6). Posaconazole is suggested as a proper agent for patients with mucormycosis undergoing surgical removal. The prognosis in patients with serious infection is better in the case of early diagnosis, combination therapy with antifungal agents, and surgical removal (5). Here, we report sino-orbital mucormycosis in a pre-diabetic female infected with COVID-19. 

## Case Presentation

The patient was a 54-year-old female who was hospitalized for 12 days infected with COVID-19. Her spiral lung and Sino-orbital computerized scan is showed in Figure 2. The RT-PCR tests were positive twice while the levels of immunoglobulin G (IgG) and immunoglobulin M (IgM) were high. Naproxen, lopinavir / ritonavir, acetaminophen interferon beta1a (12 million units every other day) and dexamethasone (8 mg for 12 days.) were administered for the patients, moreover, fluid and oxygen therapies were performed by a facial mask. She returned with a complaint of sore and red eyes three days after being discharged from one of the tertiary hospital in Tehran, Iran.

**Figure 1:**
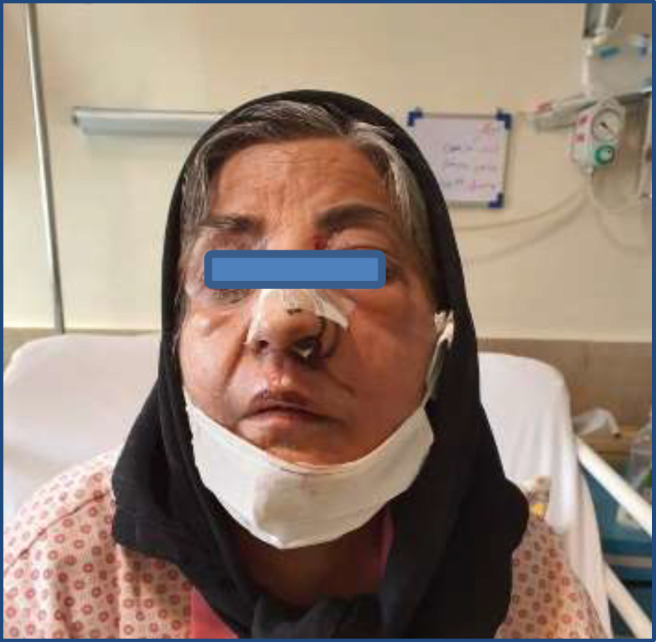
Patient's image upon admission

**Figure 2:**
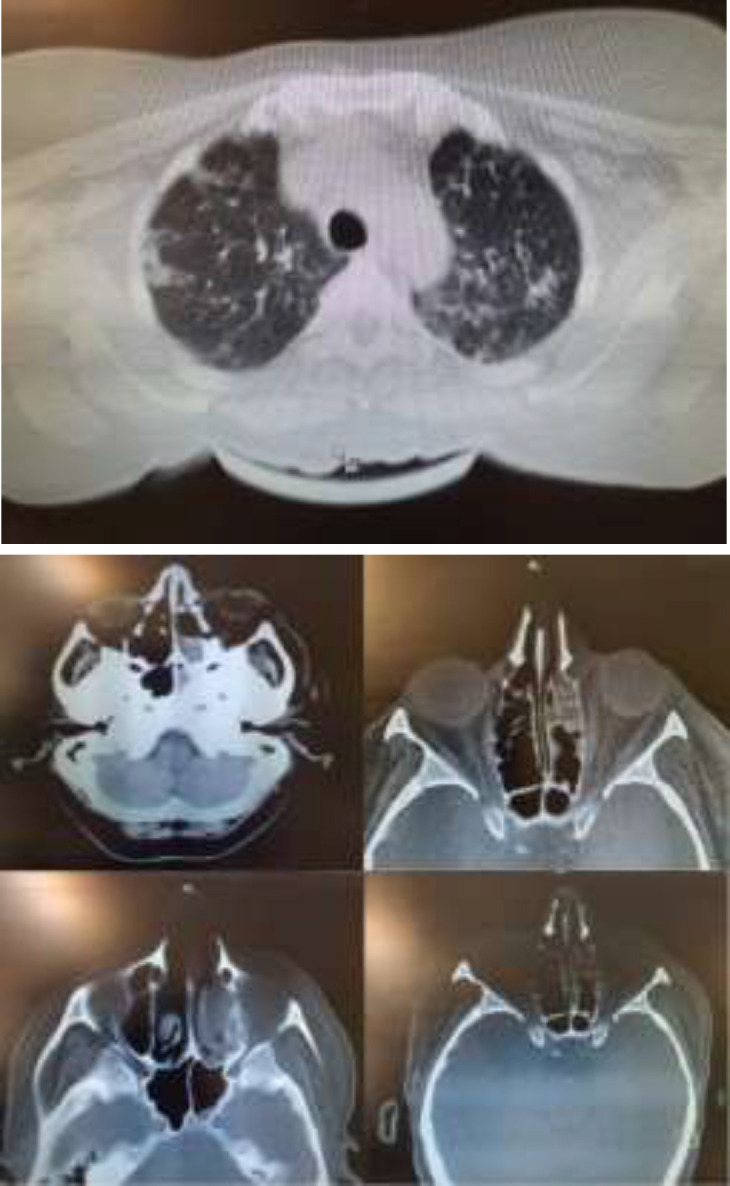
(A): Spiral chest CT scan, (B); Paranasal and periorbital sinus

A number of examinations were performed in the emergency ward as the case was prone to mucormycosis. Fortunately, the patient had no underlying problems or any signs of diabetes. The computed tomography scans of the brain, paranasal and orbital sinuses, as well as the necessary tests were performed. She was transferred to the operation room after consultation with ENT specialists and was subjected to sinus endoscopy and functional endoscopic sinus surgery (Figure 1). 

Phlegm specimens and nasal discharge were collected and sent for pathological assessments. The samples taken from the sinuses mucosa and bone tissue, Phlegm specimens and nasal discharge which was sent for pathological studies and stained with PAS, showed invasion of vascular and nervous tissue and broad, non-septate hyphae, which confirmed mucormycosis.

Table 1 presents the test results for the patient. In the predisposing factors that could be the cause of mucormycosis in this patient, undiagnosed pre-diabetic conditions and dexamethasone use were found during the previous hospitalization. 

**Table 1:** Results of the patient’s tests

**Tests**	Result
**White blood cell count **	18900(cells per cubic millimeter)
**erythrocyte sedimentation rate **	86(mm)
**C-reactive protein **	61(mg/L)
**Hemoglobin A1C**	6.3%
**Fasting blood sugar**	122(mmol/L)
**Creatinine **	1.1 mg/dL
**OGTT 2 hour blood sugar **	168 ( mg/dL)
**1,3 beta-D glucan**	Negative
**Blood culture**	Negative
**Pathology of paranasal sinuous discharge **	Fungi of the Mucorales type

**Figure 3:**
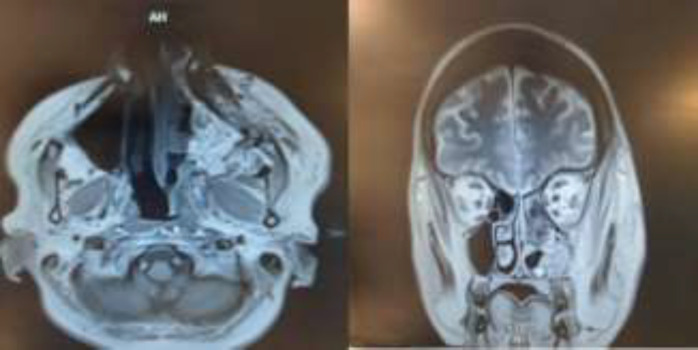
Magnetic resonance imaging findings after the intervention

Our treatment included radical surgery (Figure 3), frequent examinations of the sinuses and debridement, as well as Amphotericin B liposomal (7.5mg/kg), plasma glucose control and regular ophthalmologic examinations. After ensuring adequate surgical and pharmacological treatments, the intravenous treatment was changed to oral posaconazole (200mg /4 times daily) (overlapped for three days with injection).

After being discharged from the hospital, the patient went to the clinics for periodic examinations. There was no problem in favor of mucormycosis in the sinuses around the eye although the patient's left eye was not preserved which resulted in complete evacuation of the eye. Six month later, she referred to hospital with red eye complaint. She was admitted and more evaluations were done but no sign in favor of mucormycosis was detected.

## Discussion

Mucormycosis, one of the co-infections reported during and after COVID-19, is an invasive fungal disease which is often reported in patients with underlying diseases such as diabetes and immunodeficiency disorders. Among the fungal diseases, Aspergillosis and Mucormycosis have been reported in a number of studies (7, 8).

At the time of writing, according to the literature, there is only one case with COVID-19 and mucormycosis without underlying disorders (8). The aforementioned study reported a 33-year-old American female with COVID-19 who was diagnosed with mucormycosis and orbital compartment syndrome. Similar to our case in this study, the patient had no underlying diseases. Mucormycosis is an uncommon but deadly fungal infection that is observed on autopsy in 10% of all fungal infections which primarily affects immunocompromised patients, especially diabetics (9). This disease is characterized by infarction and necrosis of host tissues. Rhino orbital-cerebral infection is the most common clinical presentation of mucormycosis which may be secondary to the inhalation of spores into the paranasal sinuses of a susceptible host (10). It is commonly reported among patients with diabetes mellitus (70%). The majority of the patients developed ketoacidosis at the time of presentation (8). Mucormycosis may occur in patients undergoing hematopoietic stem cell transplant and those who consume steroids. Moreover, it is observed in people with neutropenia, iron overload, and malnutrition, or those who received antifungals that are not active against Mucorales (11).

The role of COVID-19 infection in the occurrence of mucormycosis is unclear. There has been no answer to the question of whether COVID-19 infection was contributory to this illness or merely coincidental. Probably, severe immunocompromised and pre-diabetic states lead to the occurrence of both mucormycosis and COVID-19. Multiple therapies, including the use of antifungal agents, surgical removal, and correction of underlying conditions that predispose the patient to disease should be considered in patients with mucormycosis. Furthermore, the control over the underlying condition is highly important in mucormycosis. Rapid correction of metabolic abnormalities in uncontrolled diabetes is also essential. If possible, corticosteroids should be discontinued and other immunosuppressive drugs should be reduced as much as possible (12).

In conclusion the most common causes of immunodeficiency are iatrogenic and almost the result of the widespread use of therapies to modulate the immune system, whether planned or haphazardly. In treating pneumonia caused by COVID-19, we have seen an increasing use of corticosteroids followed by an increased rate of side effects. Before prescribing steroids, it is important to consider patients who are in a pre-diabetic state or possibly immunocompromised. Accordingly, such patients should be considered for invasive fungal infections in the post-discharge period.
